# Sensitivity-Aware Differential Privacy for Federated Medical Imaging

**DOI:** 10.3390/s25092847

**Published:** 2025-04-30

**Authors:** Lele Zheng, Yang Cao, Masatoshi Yoshikawa, Yulong Shen, Essam A. Rashed, Kenjiro Taura, Shouhei Hanaoka, Tao Zhang

**Affiliations:** 1 School of Computer Science and Technology, Xidian University, Xi’an 710126, China; llzheng@stu.xidian.edu.cn (L.Z.); ylshen@mail.xidian.edu.cn (Y.S.); 2 Department of Computer Science, Institute of Science Tokyo, Tokyo 152-8550, Japan; cao@c.titech.ac.jp; 3 Faculty of Data Science, Osaka Seikei University, Osaka 533-0007, Japan; yoshikawa-mas@osaka-seikei.ac.jp; 4 Graduate School of Information Science, University of Hyogo, Hyogo 670-0092, Japan; rashed@gsis.u-hyogo.ac.jp; 5 Graduate School of Information Science and Technology, University of Tokyo, Tokyo 113-0033, Japan; tau@eidos.ic.i.u-tokyo.ac.jp; 6 Graduate School of Medicine, University of Tokyo, Tokyo 113-0033, Japan; hanaoka-tky@g.ecc.u-tokyo.ac.jp

**Keywords:** smart healthcare, differential privacy, gradient inversion attacks, federated learning

## Abstract

Federated learning (FL) enables collaborative model training across multiple institutions without the sharing of raw patient data, making it particularly suitable for smart healthcare applications. However, recent studies revealed that merely sharing gradients provides a false sense of security, as private information can still be inferred through gradient inversion attacks (GIAs). While differential privacy (DP) provides provable privacy guarantees, traditional DP methods apply uniform protection, leading to excessive protection for low-sensitivity data and insufficient protection for high-sensitivity data, which degrades model performance and increases privacy risks. This paper proposes a new privacy notion, sensitivity-aware differential privacy, to better balance model performance and privacy protection. Our idea is that the sensitivity of each data sample can be objectively measured using real-world attacks. To implement this new notion, we develop the corresponding defense mechanism that adjusts privacy protection levels based on the variation in the privacy leakage risks of gradient inversion attacks. Furthermore, the method extends naturally to multi-attack scenarios. Extensive experiments on real-world medical imaging datasets demonstrate that, under equivalent privacy risk, our method achieves an average performance improvement of 13.5% over state-of-the-art methods.

## 1. Introduction

Smart healthcare is transforming medicine by integrating artificial intelligence (AI) and distributed computing to improve diagnostics, treatment, and patient care [1,2,3]. Federated learning (FL) plays a key role in this transformation by enabling collaborative AI model training without sharing raw patient data [4,5]. Instead, only model updates are exchanged, preserving privacy and ensuring compliance with strict data protection regulations [6,7]. By allowing models to learn from diverse patient populations across different regions, FL fosters global collaboration, mitigates regulatory conflicts, and accelerates AI advancements in medical imaging [8,9]. Its decentralized nature ensures a broader representation of medical data, leading to more generalizable and inclusive AI models, making it a cornerstone of next-generation smart healthcare.

**Figure 1:**
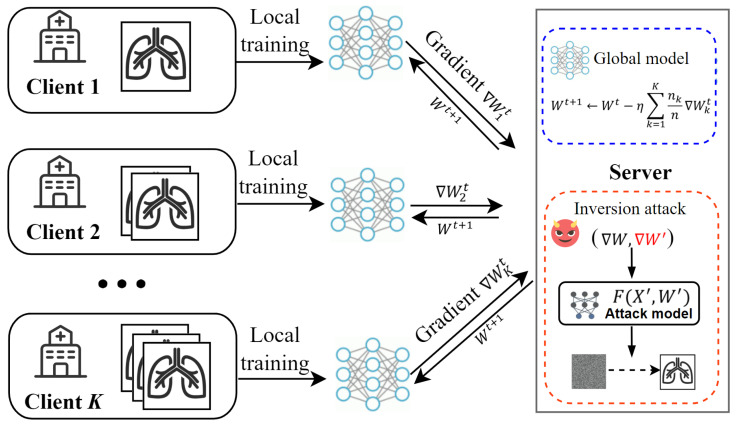
Federated learning in medical imaging.

Despite the promise of data privacy in FL, recent studies have shown that transmitting gradient updates instead of raw training samples can create a false sense of security [10,11,12,13]. As shown in Figure 1, techniques such as gradient inversion attacks exploit shared gradient information to reconstruct original medical data. These attacks highlight the potential privacy risks inherent in gradient information, exposing the limitations of federated learning when faced with sophisticated threats. To address these privacy risks, researchers have proposed various protective strategies, among which differential privacy (DP) has attracted significant attention given its formal and provable privacy guarantees [14,15,16,17]. By introducing random noise into model updates, DP makes it difficult for attackers to accurately determine whether a specific sample participated in the training process. This mechanism effectively mitigates the privacy leakage risk while offering a flexible balance between privacy protection and model performance.

However, canonical DP notions typically assume that all data are equally sensitive and apply a uniform noise level to all data. This uniform approach often results in poor model performance and can even hinder the convergence of the training process. Our research was inspired by a key observation that different data samples have different levels of privacy risks when subjected to adversarial attacks. For example, rare or anomalous data may be more vulnerable to attacks because they significantly impact model updates. Moreover, the characteristics of the attack can further exacerbate these differences in risk. For instance, gradient inversion attacks pose higher risks to samples with high gradient sensitivity, while attribute inference attacks are more likely to target prominent features within the data. These indicate that some data are more vulnerable than others, and applying uniform noise fails to mitigate the resulting uneven risks effectively. Therefore, it is essential to design more fine-grained and dynamic privacy protection strategies to protect privacy while minimizing the impact on model performance.

In this paper, we propose a novel DP notion, named sensitivity-aware differential privacy (SDP), which provides customized privacy protection for different training data while improving the predictive performance of the model. Unlike traditional differential privacy notions that provide uniform protection for all data, SDP dynamically adjusts protection levels based on the sensitivity of different data samples. Critically, we emphasize that the sensitivity of different data should be determined by their privacy leakage risks under real-world attacks rather than relying on simplistic uniform strategies or subjective user decisions, as in prior works. This is particularly important because users often lack the ability to accurately evaluate the privacy levels of their data.

To realize this new notion, we designed a privacy defense mechanism to counter the gradient inversion attacks encountered during federated medical imaging training. We chose gradient inversion attacks as the target because they are among the most prevalent in federated learning, requiring no additional assumptions from the attacker beyond access to model gradient updates, as shown in Figure 1. Specifically, before sharing gradient updates, each client locally simulates gradient inversion attacks to evaluate the sensitivity of different data. Based on the calculated sensitivity, noise is adaptively added, with higher levels of noise applied to data with a greater privacy risk, ensuring stronger protection for more vulnerable data. By addressing the limitations of uniform noise application, our method enhances privacy protection in areas of higher risk while significantly improving the overall predictive performance of the federated model. Thus, it is a promising solution for sensitive medical data scenarios. Our contributions are as follows:

1. We propose a novel sensitivity-aware differential privacy (SDP) notion that evaluates data sensitivity based on privacy risks under adversarial attacks, ensuring customized protection for individual data samples.
2. To realize this notion, we designed a privacy defense mechanism to counter the gradient inversion attacks encountered during federated medical imaging training. Our approach provides stronger protection for data with higher privacy risks, effectively balancing privacy and utility.
3. We demonstrate the natural scalability of our approach in handling multiple attack scenarios. By refining the sensitivity function, our method effectively quantifies comprehensive privacy risks and provides adaptive protection in complex threat environments.
4. Theoretical analysis and experimental results validate the effectiveness of our method. Furthermore, while this work focuses on federated medical imaging, the proposed SDP notion is broadly applicable to other domains where data samples exhibit varying levels of sensitivity to adversarial attacks.

## 2. Related Work

Federated learning, which enables cross-institutional collaborative model training without the need for centralized storage or the exchange of sensitive data, has been widely applied to various medical tasks such as brain tumor classification [18], breast density classification [19], and COVID-19 detection [20]. However, despite its advantages, FL remains vulnerable to gradient inversion attacks (GIAs), which can reconstruct sensitive training data from shared gradients [10,21,22,23]. To address these privacy risks, differential privacy (DP) has been extensively explored as a potential defense mechanism. This section reviews the prior work in two key areas: (1) the privacy risks posed by gradient inversion attacks on medical data and (2) the application of differential privacy in medical imaging and healthcare AI.

### 2.1. Gradient Inversion Attacks on Medical Data

One of the first works to demonstrate gradient leakage was Deep Leakage from Gradients (DLG) by Zhu et al. [10], which formulated the gradient inversion problem as an optimization task. The authors showed that, given only the gradient updates, an adversary can iteratively refine an image until its gradient matches the original training data, thereby reconstructing medical images with high accuracy. Later, Zhao et al. [11] improved this method with iDLG, which leverages additional label information to further enhance reconstruction quality. These studies provided early evidence that FL is susceptible to data reconstruction, contradicting the assumption that gradient updates alone do not expose sensitive information. Further research has enhanced the effectiveness of gradient inversion attacks. Geiping et al. [12] demonstrated that adding regularization constraints, such as cosine similarity, significantly improves the fidelity of reconstructed images. Their method, which aligns the adversarially generated image with the original training data in the gradient space, has been widely used as a benchmark for evaluating privacy risks in FL. More recent work by Hatamizadeh et al. [22] specifically analyzed GIA attacks in federated medical imaging. Their findings revealed that medical images, particularly those with lower structural complexity (such as grayscale X-rays), are highly vulnerable to reconstruction, reinforcing the urgency of developing stronger privacy-preserving mechanisms. Gradient-based model inversion attacks have also been studied in broader healthcare contexts. Li et al. [23] demonstrated that adversarial gradient optimization could reconstruct pathological features in medical images, raising ethical concerns about deploying FL in clinical applications. These studies collectively highlight that gradient inversion attacks are a major privacy risk for medical federated learning, necessitating the development of robust privacy defenses that go beyond simple gradient obfuscation techniques.

### 2.2. Differential Privacy for Medical Data Protection

To counteract privacy risks in federated learning (FL), differential privacy (DP) has been widely explored as a formal method to limit information leakage from gradient updates. DP achieves this by introducing random noise to gradients before they are shared, ensuring that individual data points cannot be precisely inferred. Differentially private stochastic gradient descent (DPSGD) is the most prominent mechanism for training machine learning models under DP guarantees [24,25]. It modifies standard SGD by clipping gradients to a fixed norm *C* and adding Gaussian noise scaled by σC, where σ controls the privacy budget ϵ. DPSGD has been adapted by several works to address privacy concerns in medical applications. For instance, Kaissis et al. [21] proposed PriMIA, an open-source framework that integrates DP with secure aggregation and encrypted inference for medical imaging tasks. Ziller et al. [26] presented a variant of DPSGD tailored for medical image analysis and demonstrated its feasibility on real-world datasets. However, these methods assume uniform sensitivity across all medical images and apply identical noise levels to all data samples. This results in excessive noise applied to less-sensitive data, which degrades model performance, and insufficient protection for more-sensitive samples, thereby increasing privacy risks.

Beyond the basic applications of DPSGD, several recent works have attempted to personalize DP mechanisms. Personalized DP (PDP) frameworks [27] allow users to set individual privacy budgets or customize model parameters based on local preferences or utility trade-offs. While PDP improves flexibility at the client level, it fundamentally ignores the heterogeneity in data sensitivity across samples, applying the same protection regardless of whether individual data points are vulnerable to attacks. Another recent method is selective-DP [28], which aims to assign different noise levels based on data sensitivity. Although selective-DP acknowledges that some data samples may require stronger protection than others, it relies on abstract or user-defined sensitivity metrics rather than actual attack models. Consequently, it fails to reflect real-world privacy leakage risks such as those posed by gradient inversion attacks.

In summary, while existing personalized or selective DP methods provide useful extensions of DPSGD, they either overlook intra-client data sensitivity (PDP) or lack attack-driven justifications (Selective-DP). A comparison summarizing these limitations and our proposed method is presented in Table 1. Our study addresses these critical gaps by introducing a novel privacy notion, sensitivity-aware differential privacy (SDP). Unlike prior methods, SDP explicitly quantifies the sensitivity of each data sample through simulated privacy attacks, such as GIAs, and calibrates noise accordingly. This design offers a data-driven approach to balance privacy and utility at a fine-grained level.

To empirically validate the effectiveness of our approach, we report a comparison in Table 2, based on the COVID-19 dataset under three privacy budgets (ϵ=1,5,10). We evaluated two metrics, prediction accuracy (Accuracy ↑), reflecting model utility, and the structural similarity index measure (SSIM ↓), which quantifies privacy leakage, where lower values indicate stronger protection. As shown in Table 2, our proposed SDP framework consistently outperforms the baseline methods [21,29] across all privacy levels. At ϵ=1, our method achieves an accuracy of 0.59, corresponding to a relative improvement of 40.5% over [21] (0.42) and 31.1% over [29] (0.45), while reducing the SSIM to 0.06—25% and 33.3% lower than the baseline methods, respectively. Similar trends are observed at ϵ=5 and ϵ=10, confirming the robustness of our method across privacy regimes. These results demonstrate that sensitivity-aware noise calibration yields a significantly improved privacy–utility trade-off compared to existing methods. More experimental results can be found in Section 6.

## 3. Sensitive-Aware Differential Privacy

The canonical DP notion treats all records as equally sensitive. Previous studies have explored variants of DP, such as personalized DP [27], one-sided DP [30], and selective DP [28], to account for varying privacy levels among records. While these approaches introduce flexibility in privacy allocation, they typically assume that the privacy levels of different records are predetermined or subjectively assigned by users. This assumption is often unrealistic in many practical scenarios, as users may lack the expertise to accurately assess the privacy sensitivity of their data. Moreover, privacy risks are not static but can vary depending on the attack methodology, dataset properties, and adversarial capabilities. For example, gradient inversion attacks pose a higher threat to certain medical images based on their structural complexity, yet traditional DP frameworks fail to account for such variations. Instead of relying on subjective or predefined privacy levels, an effective privacy mechanism should be adaptive and responsive to actual privacy risks.

Problem formulation: In federated learning, consider a system with *K* clients, where each client *k* has a local dataset $D_{k}={\left\{x_{i}^{\left(k\right)}\right\}}_{i=1}^{n_{k}}$. We assume the presence of a potential privacy attack function *f*, which aims to reconstruct sensitive input data from observed gradients. As a concrete example, we consider gradient inversion attacks (GIAs), where adversaries leverage optimization techniques to reconstruct private inputs from model updates. Our objective was to design a perturbation mechanism M that adaptively adds noise $\epsilon_{i}^{\left(k\right)}$ to the gradient of each data sample based on its estimated sensitivity in order to achieve the following goals:

1. Privacy guarantee : for each client *k*, mechanism M must satisfy $\left(\epsilon_{k},\delta_{k}\right)$-differential privacy.
2. Utility preservation: under the given privacy constraints, the mechanism should minimize the negative impact of the added noise on model utility, thereby maintaining high global model performance.

To address this problem, we developed sensitivity-aware differential privacy (SDP), a novel privacy framework that objectively quantifies the sensitivity of each data sample using a sensitivity function. Unlike prior methods, our approach measures privacy risks dynamically by evaluating how susceptible each data sample is to real-world adversarial attacks, particularly gradient inversion attacks. Based on this sensitivity assessment, SDP applies a customized privacy budget allocation, ensuring that data points with higher privacy risks receive stronger noise protection, while better utility is maintained for those with lower risks. This adaptive strategy enhances privacy preservation while minimizing unnecessary model performance degradation, making it particularly well-suited for privacy-sensitive applications such as federated medical imaging.

**Definition** **1** (Sensitivity Function). *Given a privacy attack method f and a user’s local dataset D, the sensitivity function $R_{f}:D\to \left[0,1\right]$ quantifies the vulnerability of any data sample $x_{i}\in D$ under attack f. Specifically, the value of $R_{f}\left(x_{i}\right)$ reflects the sensitivity of $x_{i}$ when facing attack f. The sensitivity function is defined as*

(1) $$R_{f}\left(x_{i}\right)=h\left(Metric\left(f\left(x_{i}\right),x_{i}\right)\right),$$

where $f(x_{i})$ represents the output of the attack function *f* applied to sample $x_{i}$, such as reconstructed data. The function Metric(·,·) evaluates the effectiveness of attack *f* on $x_{i}$, using metrics like the structural similarity index measure (SSIM) or Euclidean distance. h(·) is a monotonic mapping function that translates the metric value into a normalized sensitivity score. The resulting value $R_{f}\left(x_{i}\right)$ indicates the sensitivity of sample $x_{i}$, with higher values corresponding to greater vulnerability under attack *f*.

**Definition** **2** (Sensitive-Aware Differential Privacy). *Given a sensitivity function $R_{f}$, a randomized algorithm M:D→R satisfies $\left(R_{f},\epsilon ,\delta \right)$-sensitivity-aware differential privacy if, for any two neighboring datasets D and $D^{\prime }$ and any output S⊆R,*

(2) $$\mathrm{Pr}\left[M\left(D\right)\subseteq S\right]\leq e^{\epsilon_{i}}\mathrm{Pr}\left[M\left(D^{\prime }\right)\subseteq S\right]+\delta_{i},$$

where $\epsilon_{i}=\epsilon \cdot \phi \left(R_{f}\left(x_{i}\right)\right)$ is the dynamically allocated privacy budget, and $\delta_{i}=\delta \cdot \phi \left(R_{f}\left(x_{i}\right)\right)$ is the dynamically allocated probability relaxation term. ϕ(·) is a monotonic mapping function that ensures higher sensitivity corresponds to stricter privacy protection (i.e., smaller effective $\epsilon_{i}$ and $\delta_{i}$). Essentially, SDP provides indistinguishability similar to standard DP, but it targets different sensitivity levels for different records.

**Definition** **3** (**Sequential Composition of LDP** [**31**]). *If randomized algorithm $M_{i}:X\to \mathrm{range}\left(M_{i}\right)$ satisfies $\epsilon_{i}$-LDP for i=1,2,⋯,n, then the sequential combination of these algorithms $M_{i}\left(1\leq i\leq n\right)$ satisfies $\left(\sum \epsilon_{i}\right)$-LDP.*

## 4. Sensitivity-Aware Privacy Mechanism

We propose a privacy defense mechanism for federated medical imaging training to address gradient inversion attacks based on the new sensitivity-aware differential privacy (SDP) notion. Gradient inversion attacks were selected as the focus because they were the most prevalent in this context, relying solely on gradient updates without requiring additional assumptions. Privacy mechanisms typically protect data by introducing noise into the model, such as Laplace noise (Laplace mechanism) or Gaussian noise (Gaussian mechanism). Abadi et al. [24] introduced DPSGD, which adds Gaussian noise to gradients and employs stochastic gradient descent (SGD) to train private deep learning models. In this work, we developed sensitivity-aware DPSGD to counter gradient inversion attacks and achieve SDP, as shown in Algorithm 1. Our approach consists of two key steps: sensitivity measurement and sensitivity-based noise addition. The sensitivity measurement step evaluates the privacy risks faced by different data samples under attack, while the noise addition step adjusts the noise proportionally based on the measured sensitivity.
**Algorithm 1** Sensitivity-aware privacy mechanism.**Require:**ϵ: the total privacy budget, $n_{k}$: number of images for client *k*, $x_{k}^{t}$: input data  for user *k* in round *t*, *C*: gradient norm bound. **Ensure:** Model parameters $W_{k}^{\hat{t}+1}$. 1: **for**i=1 to $n_{k}$ **do** 2:   Reconstruct data $x_{i}^{\prime }$ = **GIA**($\nabla W,\nabla W^{\prime }$). 3:   Calculate SSIM($x_{i},x_{i}^{\prime }$) for each image based on Equation (4). 4: **end for** 5: **for** i=1 to $n_{k}$ **do** 6:   Calculate the sensitivity $R_{f}\left(x_{i}\right)$ of each image $x_{i}$ based on Equation (5). 7:   Allocate privacy budget according to Equation (6). 8: **end for** 9: $W_{k}^{t+1}\leftarrow$ **LocalTraining**($W_{k}^{t},x_{k}^{t}$) 10: $W_{k}^{\hat{t}+1}=\mathrm{DPSGD}\left(W_{k}^{t},\epsilon \left(x_{k}^{t}\right),C\right)$ 11: **Return**$W_{k}^{\hat{t}+1}$ to the server.

To facilitate understanding, we first provide a high-level overview of Algorithm 1. This algorithm implements our proposed sensitivity-aware privacy mechanism. Given each client’s local dataset and a global privacy budget, it outputs privatized model parameters by dynamically adjusting the noise according to the sensitivity of each training sample. The algorithm proceeds in two main stages. First, each data sample is locally reconstructed using a simulated gradient inversion attack to assess its privacy risk (Algorithm 1, lines 1–4). The similarity between the original and reconstructed samples, measured by the SSIM, is used to compute a normalized sensitivity score (line 6), which determines how the global privacy budget is distributed across samples (line 7). In the second stage, local model training is performed using the standard SGD algorithm (line 9), and a customized differentially private mechanism is applied, where gradients are clipped and perturbed using noise scaled inversely to each sample’s allocated privacy budget (line 10). The privatized model parameters are then returned to the server (line 11).

### 4.1. Gradient Inversion Attack in Medical Imaging

In federated learning (FL), the server is often assumed to be honest but curious, meaning that while it correctly follows the aggregation protocol and facilitates iterative training across multiple clients, it may also attempt to extract sensitive information from users’ private training data, such as patient medical imaging. Unlike traditional centralized learning, where raw data are directly shared, FL relies on gradient updates to train models collaboratively. However, these gradient updates can still leak private information, making them a target for adversarial attacks. One of the most effective methods for exploiting this vulnerability is the gradient inversion attack (GIA), which aims to reconstruct the original training samples by analyzing shared gradients. The FL server, acting as an adversary, can systematically apply a GIA to extract private training data from target users without requiring direct access to their raw data. This type of attack is particularly concerning in medical imaging applications, where leaked data can expose sensitive patient diagnoses, clinical records, and personal health information. The workflow of a gradient inversion attack follows several key steps:

1. Dummy data initialization: The FL server, acting as an adversary, begins by randomly generating a pair of dummy input data and a corresponding label. These dummy values are iteratively refined to match the gradients of the original training data.
2. Gradient capture: The server collects the gradient updates submitted by honest clients during each training iteration. Since FL operates in a distributed manner, clients compute gradients locally on their private datasets before sending them to the server. These gradients contain valuable information about the underlying training samples, making them susceptible to reconstruction.
3. Gradient-matching optimization: The adversary then employs an optimization process to iteratively adjust the dummy data and labels, aiming to minimize the difference between the true gradients (received from clients) and the gradients computed from the dummy data. The most common method for calculating the difference between gradients is
  (3) $$∥\nabla W^{\prime }{-\nabla W∥}^{2}={∥\frac{\partial \ell (F\left(x^{\prime },W\right),y^{\prime })}{\partial W}-\nabla W∥}^{2},$$
  where $x^{\prime }$ and $y^{\prime }$ are the data and their label, respectively; ∇W and $\nabla W^{\prime }$ are the true and dummy gradients, respectively.
4. Iterative refinement: The gradient-matching process is executed iteratively, with the attacker continuously adjusting the dummy data to minimize the gradient difference. This optimization continues until one of two stopping conditions is met: (1) the loss function begins to diverge, suggesting that further refinement will not yield a more accurate reconstruction, or (2) the maximum number of iterations is reached, at which point the attacker achieves a high-fidelity approximation of the original training sample.

Gradient inversion attacks leverage the fact that gradient updates preserve the structural information about the input data. This means that for high-resolution medical images, such as chest X-rays, MRI scans, or retinal fundus images, the reconstructed samples can retain fine-grained details, making it possible to identify patient conditions or clinical features. Furthermore, medical imaging datasets often contain structured patterns, such as organ contours or lesion textures, which facilitate high-quality reconstruction compared to natural images. Beyond the standard gradient-matching approach, advanced variations of the GIA incorporate regularization techniques and prior knowledge to further improve attack efficiency. For example, Geiping et al. [12] introduced cosine similarity-based optimization to enhance reconstruction accuracy, while Hatamizadeh et al. [22] demonstrated that medical imaging models are particularly vulnerable due to their reliance on fine-grained features.

### 4.2. Sensitivity Measurement

During the model training process, the server can launch gradient inversion attacks using only the gradients uploaded by clients to reconstruct the original data. In other words, gradients are the only information required by the attacker. Therefore, clients can locally perform gradient inversion attacks using the model parameters about to be uploaded to simulate the reconstruction of the original data, as illustrated on line 2 of Algorithm 1. By simulating the reconstruction process of an attacker, clients can evaluate the privacy leakage risk of each training sample. The structural similarity index measure (SSIM) is commonly used to calculate the similarity between reconstructed images and original images. Thus, the SSIM can naturally serve as a metric for quantifying the privacy risk levels of different data samples facing gradient inversion attacks. The metric is defined as follows:

(4) $$\mathrm{SSIM}\left(x_{i},x_{i}^{\prime }\right)=\frac{\left(2\mu_{x_{i}}\mu_{x_{i}^{\prime }}+C_{1}\right)\left(2\sigma_{x_{i}x_{i}^{\prime }}+C_{2}\right)}{\left(\mu_{x_{i}}^{2}+\mu_{x_{i}^{\prime }}^{2}+C_{1}\right)\left(\sigma_{x_{i}}^{2}+\sigma_{x_{i}^{\prime }}^{2}+C_{2}\right)},$$

where $\mu_{x_{i}}$ and $\mu_{x_{i}^{\prime }}$ are the mean intensities of images $\mu_{x_{i}}$ and $\mu_{x_{i}^{\prime }}$, respectively. $\sigma_{x_{i}}^{2}$ and $\sigma_{x_{i}^{\prime }}^{2}$ are the variances (contrast) of images $\mu_{x_{i}}$ and $\mu_{x_{i}^{\prime }}$. $\sigma_{x_{i}x_{i}^{\prime }}$ is the covariance (structural similarity) between images $\mu_{x_{i}}$ and $\mu_{x_{i}^{\prime }}$. $C_{1}$ and $C_{2}$ are small constants that stabilize the division, avoiding instability when the denominator is close to zero.

By calculating the SSIM for each medical image, clients can effectively evaluate the privacy sensitivity of different images. A higher SSIM value indicates greater similarity between the reconstructed and original images, reflecting higher reconstruction quality. This, in turn, suggests a greater privacy risk, as an attacker can extract more detailed information from the reconstructed image. Consequently, the higher the SSIM value, the higher the privacy sensitivity of the data. Building on this observation, we quantify the sensitivity of each data sample based on its SSIM value. Specifically, the sensitivity is determined:

(5) $$R_{f}\left(x_{i}\right)=\frac{\mathrm{SSIM}(x_{i},x_{i}^{\prime })}{\sum_{j=1}^{n_{k}}\mathrm{SSIM}\left(x_{j},x_{j}^{\prime }\right)},$$

where $n_{k}$ is the number of medical images of client *k*.

For illustration, consider a local dataset with three samples. After simulating gradient inversion attacks, the SSIM values between original and reconstructed images are computed as 0.88, 0.65, and 0.47. These values are normalized to obtain the sensitivity scores using Equation (5): 0.40, 0.30, and 0.21, respectively. Based on these scores, the privacy budget is distributed according to Equation (6), where samples with higher sensitivity (e.g., SSIM = 0.88) receive stronger noise (i.e., smaller $\epsilon_{i}$), ensuring enhanced protection for vulnerable data.

### 4.3. Sensitivity-Based Noise Addition

Using the SSIM as a measurement metric, we evaluated the privacy sensitivity of different data samples. Our experiments in Section 6 also show that different data samples exhibit varying privacy risks under identical gradient inversion attack settings. Thus, we propose adding customized noise levels based on the specific privacy sensitivity of each data sample. Specifically, we calculate the privacy budget for each data sample based on the sensitivity and then add the corresponding noise. The privacy budget $\epsilon_{i}$ for each data sample $x_{i}$ is determined:

(6) $$\epsilon_{i}=\frac{\epsilon \cdot \exp(-\alpha R_{f}\left(x_{i}\right))}{\sum_{j}\exp\left(-\alpha R_{f}\left(x_{j}\right)\right)},$$

where $R_{f}\left(x_{i}\right)$ represents the privacy sensitivity of $x_{i}$ under gradient inversion attacks, ϵ represents the total privacy budget, and α is an adjustment parameter used to control the relationship between sensitivity and the privacy budget. Once the privacy budget for each data sample is determined, we apply a dynamic DPSGD algorithm to perturb the gradients $g_{i}$ before they are uploaded. The algorithm begins by clipping each sample’s gradient to limit its sensitivity, normalizing gradient *g* to a predefined threshold *C*. Gaussian noise is then added to the gradient based on the allocated privacy budget, ensuring that data with higher sensitivity receive stronger noise protection, while data with lower sensitivity experience less perturbation. Specifically,

(7) $$\begin{array}{cc} g_{i}^{\prime } & =\frac{g_{i}}{\max(1,∥g_{i}{∥}_{2}/C)}, \end{array}$$

(8) $$\begin{array}{cc} \;\;\tilde{g_{i}} & =g_{i}^{\prime }+N\left(0,\sigma_{i}^{2}C^{2}I\right),\sigma_{i}\propto \frac{1}{\epsilon_{i}} \end{array}$$

where *C* is the clipping threshold, and $\sigma_{i}$ is the noise scale. This adaptive approach ensures that data with higher privacy risks receive stronger protection, while higher prediction accuracy is retained for data with lower sensitivity.

**Figure 2:**
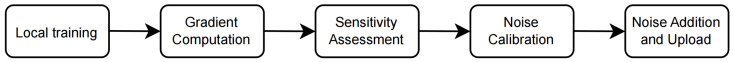
Workflow of sensitivity-aware privacy mechanism.

Figure 2 shows the workflow of the sensitivity-aware privacy mechanism. Each client, such as a medical institution, begins by training a local model using its private dataset. After training, the client computes gradients from its model parameters. To evaluate the potential privacy risk, the client performs a simulated gradient inversion attack (GIA) on its computed gradients. This step assesses how susceptible each data sample is to reconstruction, providing a quantifiable measure of sensitivity. Based on this sensitivity assessment, the framework calibrates the amount of noise to be added to each gradient. Data samples with higher sensitivity receive more noise, while those with lower sensitivity receive less, ensuring a tailored privacy protection approach. The calibrated noise is then added to the gradients, and these differentially private updates are transmitted to the central server. This adaptive mechanism ensures that privacy protection is proportionate to the actual risk of data leakage, optimizing the balance between data utility and privacy.

### 4.4. Theoretical Analysis

#### 4.4.1. Privacy Guarantee

Given a sensitivity function $R_{f}$ that quantifies the sensitivity of each data sample $x_{i}$, the privacy budget for $x_{i}$ is dynamically allocated as $\epsilon_{i}=\epsilon \cdot \phi \left(R_{f}\left(x_{i}\right)\right),$ where ϕ(·) is a mapping function. Using the dynamic DPSGD mechanism, noise is added to each sample’s clipped gradient $g_{i}$ with a Gaussian noise scale of $\sigma_{i}\propto \frac{1}{\epsilon_{i}}$, which guarantees that each data sample $x_{i}$ independently satisfies $\left(\epsilon_{i},\delta_{i}\right)$-DP. Here, $\delta_{i}$ is proportionally allocated as $\delta_{i}=\delta \cdot \phi \left(R_{f}\left(x_{i}\right)\right),$ with the property that $\sum_{i}\delta_{i}=\delta$. According to the sequential composition theorem of LDP, if each data sample satisfies $\left(\epsilon_{i},\delta_{i}\right)$-LDP, the overall mechanism satisfies $(\sum_{i}\epsilon_{i},\sum_{i}\delta_{i})\text{-}\mathrm{LDP}$. Since the total privacy budget satisfies $\sum_{i}\epsilon_{i}=\epsilon$ and $\sum_{i}\delta_{i}=\delta$, the proposed algorithm satisfies (ϵ,δ)-LDP, ensuring that the entire system adheres to the desired differential privacy guarantee.

#### 4.4.2. Computational Overhead

We employ a standard gradient inversion attack method locally on each client, leveraging gradient information and optimization algorithms to reconstruct data without requiring additional model training, making the approach computationally efficient. This simulation is conducted offline on the client’s device prior to training and does not interfere with the training process. The overall computational overhead is determined solely by the number of local samples on each client, where the computational complexity is O(n), with *n* denoting the number of local data samples. Moreover, each client only needs to evaluate the sensitivity of its own data once before the start of federated training. By offloading the sensitivity assessment to a pre-training step, our method eliminates the need for repeated computations and significantly reduces the burden during the actual training process. As a result, increasing the number of clients does not introduce additional computational or communication overhead during training, and the scalability of our method remains comparable to that of traditional federated learning approaches. This design ensures that our sensitivity-aware differential privacy (SDP) framework is both computationally efficient and practically feasible for real-world FL deployments.

#### 4.4.3. Communication Overhead

The current methods involve transmitting model updates of size d from each client to the server in every communication round, resulting in a communication complexity of O(d) per client per round. Our method maintains the same communication pattern as existing methods, with each client transmitting model updates of size *d* per round. However, due to the sensitivity-aware noise addition, our method may achieve convergence with fewer communication rounds, potentially reducing the overall communication cost.

## 5. Multi-Attack Sensitivity Evaluation

In practical FL scenarios, clients encounter various privacy threats rather than a single type of attack. These attacks have different targets and methods but pose significant data privacy risks. For example, gradient inversion attacks aim to reconstruct the original data, making samples with high gradient sensitivity particularly vulnerable. Attribute inference attacks tend to exploit samples with prominent features to infer sensitive attributes. Existing DP methods usually either apply uniform noise across all data or focus on mitigating a single type of attack. However, these approaches fail to address the combined risks of multiple concurrent attacks, resulting in significant limitations in multi-attack scenarios. On the one hand, the superposition of different attacks may exacerbate the cumulative risk of certain data samples. On the other hand, current methods lack the ability to dynamically adjust protection levels, making it difficult to balance the privacy of high-risk samples with the overall utility of the model.

Our method seamlessly extends to scenarios where clients encounter multiple types of attacks. The central idea is to integrate the impacts of various attacks during the sensitivity evaluation phase. By employing a weighted approach, the sensitivities of individual attacks are combined into a unified, comprehensive sensitivity metric. This metric is then used to dynamically allocate the privacy budget, ensuring effective privacy protection in various attack scenarios. Specifically, assume that the client faces *m* potential attacks, each described by an attack function $f_{j}$ (j=1,2,…,m). For a given data sample $x_{i}$, the sensitivity under attack $f_{j}$ is defined as $R_{f_{j}}\left(x_{i}\right)$, which represents the privacy leakage risk posed by the specific attack. The overall sensitivity $R_{f}\left(x_{i}\right)$, reflecting the cumulative privacy risk across all attacks, is calculated as a weighted sum:

(9) $$R_{f}\left(x_{i}\right)=\sum_{j=1}^{k}w_{j}\cdot R_{f_{j}}\left(x_{i}\right),$$

where $w_{j}$ denotes the weight of attack $f_{j}$, representing its relative importance or threat level. The weights $w_{j}$ can be flexibly adjusted based on the specific application scenario, such as the likelihood, severity, or priority of each attack. This comprehensive sensitivity provides a basis for dynamically allocating privacy budgets, ensuring stronger protection for high-risk samples while reducing unnecessary interference for low-risk samples. Our core mechanism remains intact, requiring only modification of the sensitivity function definition to accommodate any multi-attack context.

In practice, the weights assigned to different attack types in the multi-attack sensitivity function can be determined through various strategies. One approach involves manually setting the weights based on prior knowledge of the relative severity or likelihood of each attack. Alternatively, the weights can be adaptively learned during training by monitoring the effectiveness of each attack type and adjusting the weights to prioritize defenses against more successful attacks. This adaptive weighting enhances the robustness of the privacy mechanism by dynamically focusing on the most threatening attack vectors. This extension highlights the flexibility and efficiency of our method, offering a practical and reliable privacy protection solution for federated learning tasks in complex threat environments.

## 6. Experiments

### 6.1. Experimental Setup

**Parameter settings.** We simulated a medical image classification task in an FL environment with 10 clients. The local training model was based on the ResNet-18 architecture, and each client employed the SGD optimizer with a learning rate of 0.01. For a clear comparison of defense method performance, we set the batch size to 1, thereby maximizing the attacker’s potential effectiveness.

**System Configuration.** All experiments were conducted on a machine equipped with an Intel Core i9-12900K CPU (16 cores, Intel Corporation, Santa Clara, CA, USA) and an NVIDIA RTX 3090 GPU (NVIDIA Corporation, Santa Clara, CA, USA). We used Python 3.10 with PyTorch 1.13.1 for model implementation and training. The federated learning simulations were managed using the Flower framework, which facilitated the orchestration of client-server interactions. To emulate a federated learning environment, we simulated multiple clients on the same machine. Each client ran in a separate process, maintaining its own local dataset and model instance.

**Datasets.** We evaluated the performance of different defense methods using two datasets, the COVID-19 dataset [32] and the Rotterdam EyePACS AIROGS (REA) dataset [33]. The COVID-19 dataset consists of publicly available chest X-ray images from both healthy individuals and patients diagnosed with COVID-19. The Rotterdam EyePACS AIROGS dataset contains high-quality retinal fundus images, primarily used for training and evaluating AI models for glaucoma risk prediction.

**Baseline.** We compared our sensitivity-aware differential privacy (SDP) method with two state-of-the-art defense techniques, PRiMIA [21] and MSDP [29], on a federated medical imaging task.

- PRiMIA is an open-source framework designed for end-to-end privacy-preserving deep learning in multi-institutional medical imaging settings. It integrates differential privacy mechanisms with secure aggregation and encrypted inference to protect patient data during model training and deployment. Specifically, PRiMIA employs differentially private stochastic gradient descent (DP-SGD) to ensure that individual data contributions remain confidential, while secure multi-party computation techniques are used to aggregate model updates without exposing raw data. This framework has the ability to maintain model performance comparable to non-private training methods while providing robust privacy guarantees against gradient-based attacks.
- MSDP is a federated learning approach tailored to handle non-independent and identically distributed (non-IID) data across multiple medical institutions. It extends the DP-SGD algorithm by incorporating mechanisms that account for data heterogeneity among clients. This method is effective in maintaining model accuracy in scenarios where data distributions vary significantly across participating sites.

### 6.2. Experimental Evaluation

#### 6.2.1. Results of the GIA Attack

To systematically evaluate the vulnerability of different datasets to gradient inversion attacks (GIAs), we randomly selected 100 data samples from each dataset and applied the attack method proposed by Hatamizadeh et al. [22] to reconstruct the original data. This work utilizes gradient updates and batch normalization statistics to reconstruct training data in federated learning, aiming to reveal privacy vulnerabilities under realistic client-side training settings. We quantified privacy leakage by reporting the SSIM scores of 100 individual samples from each dataset under GIA attacks. The SSIM was used to evaluate the similarity between the reconstructed image and the original input, where a higher score indicates greater privacy leakage.

**Figure 3:**
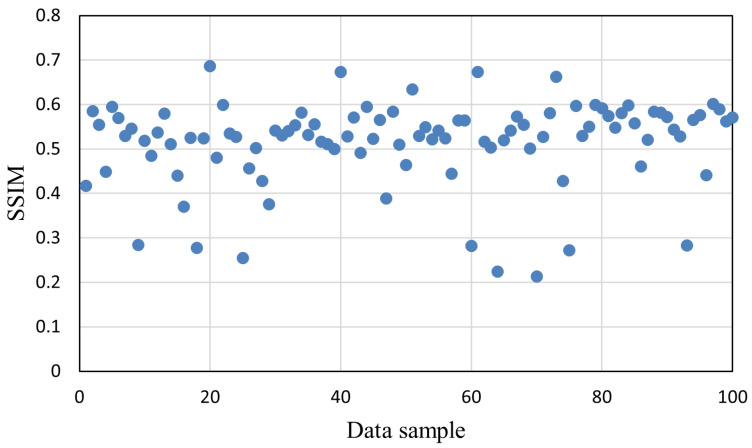
Attack results on the COVID-19 dataset.

As shown in Figure 3, the SSIM values for the COVID-19 dataset are consistently high across most samples, clustering around 0.5 to 0.6. Several samples even exceed 0.65, indicating that the attacker is able to reconstruct highly similar images in many cases. This confirms the high vulnerability of COVID-19 data to gradient inversion attacks. The relatively narrow range of SSIM scores further suggests low variability in resistance among the samples within this dataset.

**Figure 4:**
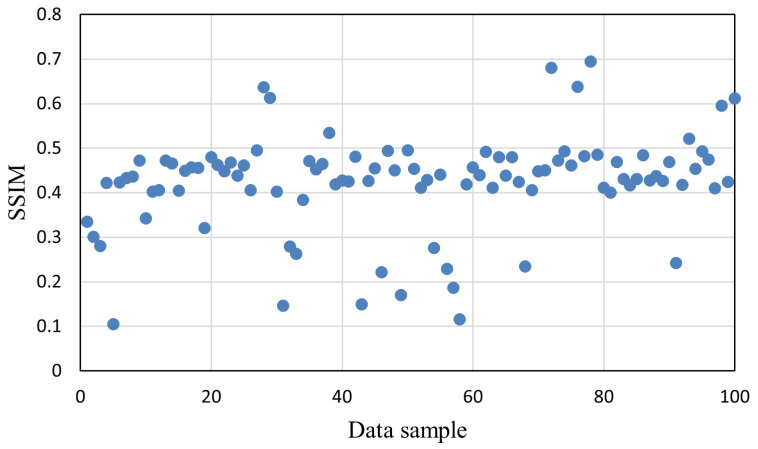
Attack results on the REA dataset.

In contrast, Figure 4 presents the SSIM distribution for the REA dataset. The values are generally lower, with a wider spread ranging from below 0.1 to just above 0.6. A significant number of samples fall below 0.3, indicating limited reconstruction success. This highlights the stronger resilience of REA data to gradient-based attacks, likely due to their higher image complexity, color channels, and feature diversity. Unlike the COVID-19 dataset, the REA samples exhibit greater variability in vulnerability, suggesting that individual data properties (e.g., texture richness or local contrast) may influence leakage risk more significantly. Taken together, Figure 3 and Figure 4 provide clear empirical evidence that the degree of privacy leakage under GIAs is highly dataset-dependent. These findings reinforce the need for adaptive privacy mechanisms that can adjust the protection levels based on the inherent vulnerability of each dataset or even individual samples.

#### 6.2.2. Results of the Sensitivity-Aware Privacy Mechanism

**Figure 5:**
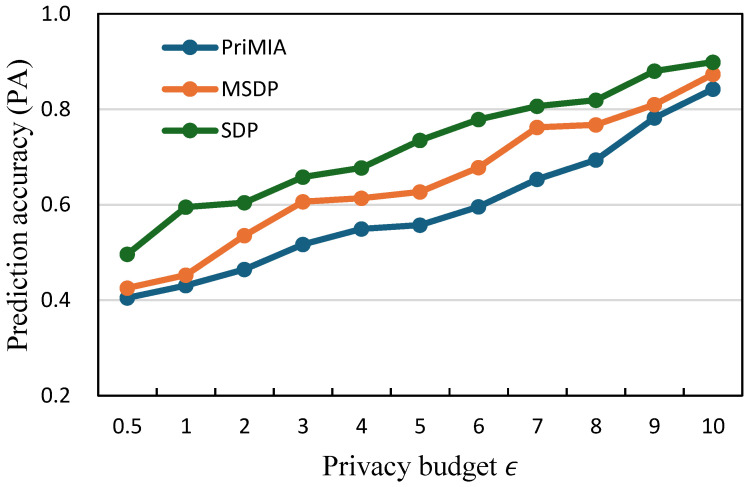
The relationship between prediction accuracy and privacy budget (COVID-19 dataset).

**Figure 6:**
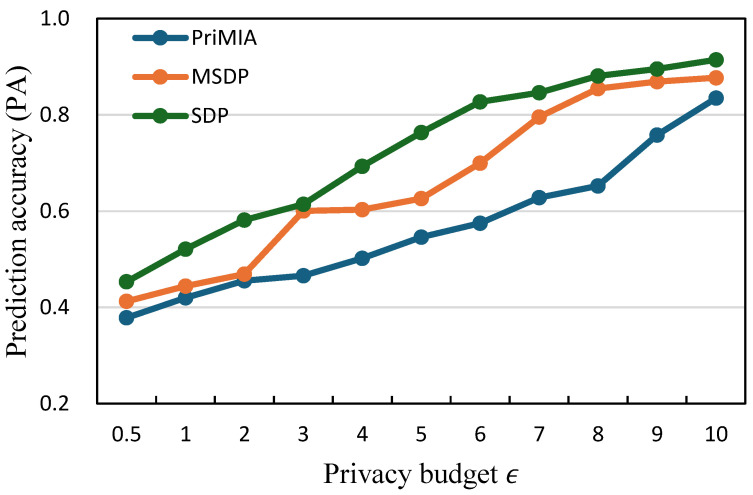
The relationship between prediction accuracy and privacy budget (REA dataset).

**The relationship between prediction accuracy and privacy budget.** We investigate how varying privacy budgets influence model performance by measuring the prediction accuracy (PA) of three representative methods: PriMIA, MSDP, and our proposed SDP. The results are presented in Figure 5 for the COVID-19 dataset and Figure 6 for the REA dataset. In both figures, the x-axis represents the privacy budget ϵ, and the y-axis reports the corresponding prediction accuracy.

In Figure 5, we can observe that as the privacy budget increases, all three methods achieve progressively higher accuracy. This trend is consistent with the intuition that a larger ϵ permits less noise to be added, thereby preserving more gradient information during model updates. Across all privacy levels, our SDP method consistently outperforms the baseline methods. For instance, at ϵ=3, SDP achieves an accuracy of approximately 0.68, whereas MSDP and PriMIA reach only 0.61 and 0.53, respectively. The performance gap is even more evident in low-budget settings such as ϵ=1, where accurate learning is more difficult due to stronger privacy constraints. A similar pattern is evident in Figure 6, which shows the results on the REA dataset. While all methods benefit from increased privacy budgets, SDP achieves the highest accuracy. At ϵ=5, for example, SDP reaches an accuracy close to 0.83, while MSDP remains below 0.75 and PriMIA under 0.60. The advantage of SDP is particularly significant in the lower ϵ regime, where accurate learning becomes more challenging. These results confirm that our sensitivity-aware differential privacy mechanism achieves a more effective privacy-utility trade-off than existing approaches. By calibrating the noise level according to the estimated sensitivity of each data sample, SDP mitigates performance degradation on less sensitive data while maintaining strong protection for more vulnerable instances. This adaptivity results in consistently improved model accuracy under a wide range of privacy budgets.

**Figure 7:**
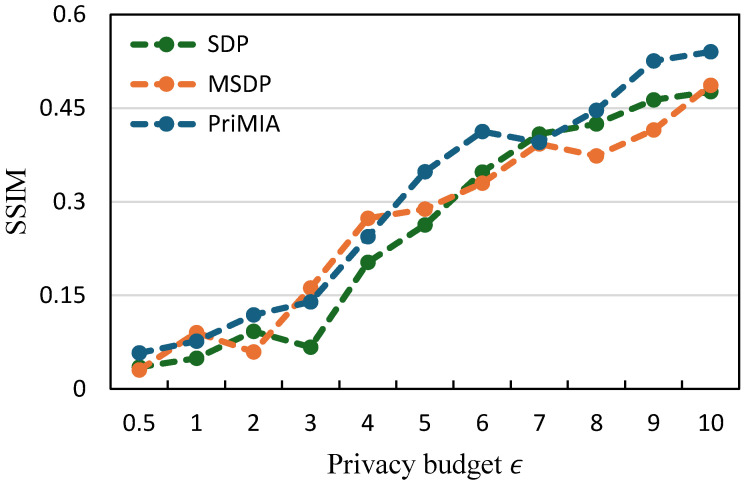
The relationship between SSIM and privacy budget (COVID-19 dataset).

**Figure 8:**
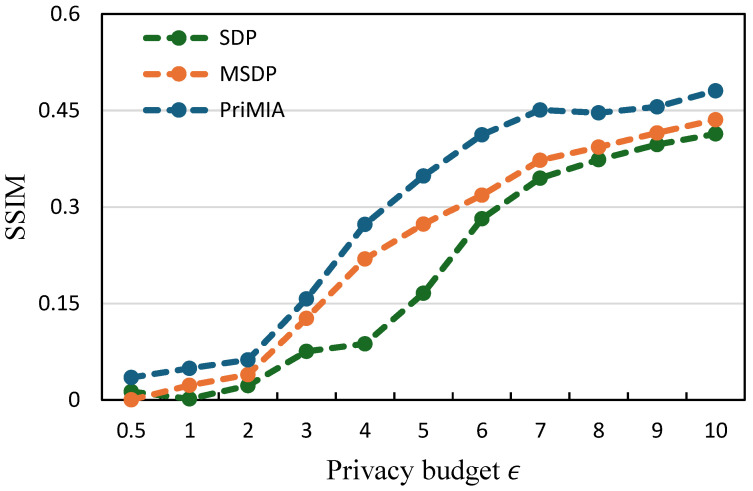
The relationship between SSIM and privacy budget (REA dataset).

**The relationship between SSIM and privacy budget.**Figure 7 and Figure 8 illustrate the average structural similarity index measure (SSIM) values of 100 reconstructed data samples under varying privacy budgets, serving as a metric of privacy leakage. The SSIM quantifies the similarity between original and reconstructed images, with higher values indicating greater potential for privacy breaches. On both datasets, an increase in the privacy budget correlates with elevated SSIM values for all methods, reflecting the trade-off between privacy protection and model utility. Importantly, our SDP method demonstrates SSIM trends comparable to, or slightly better than, those of traditional DP approaches, suggesting that adaptive noise allocation does not compromise privacy safeguards. These findings underscore the efficacy of our SDP framework in achieving a favorable balance between maintaining robust privacy protections and enhancing model performance.

**Figure 9:**
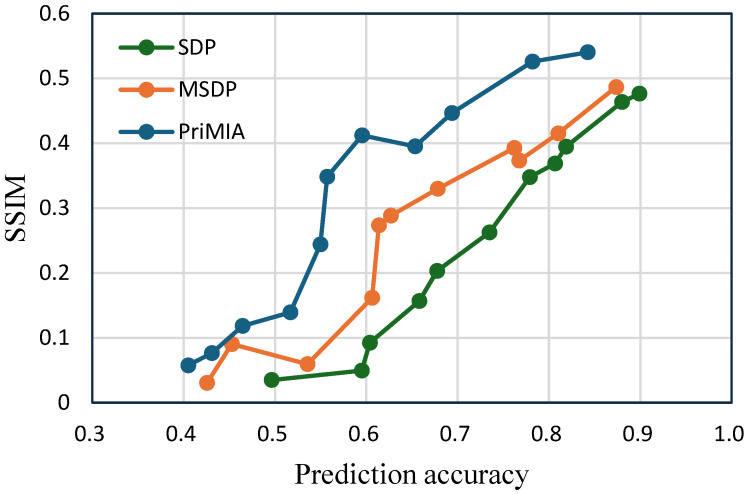
The trade-off between accuracy and privacy (i.e., SSIM). The higher the SSIM, the higher the privacy leakage, since the attacker can reconstruct high-quality images (COVID-19 dataset).

**Figure 10:**
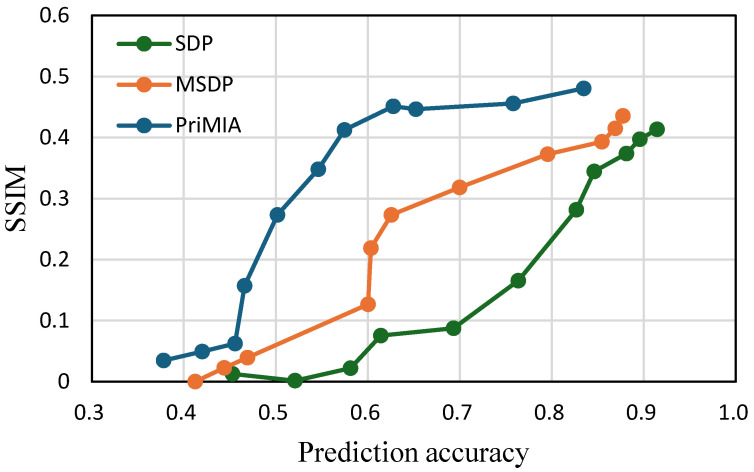
The trade-off between accuracy and privacy (REA dataset).

**The trade-off between accuracy and privacy.** To directly analyze the privacy–utility trade-off, we jointly visualize the relationship between prediction accuracy and SSIM in Figure 9 (COVID-19 dataset) and Figure 10 (REA dataset). In both plots, each point corresponds to a specific privacy budget setting, with accuracy representing model utility and the SSIM reflecting privacy leakage. An ideal method would appear in the bottom-right region of the graph, indicating high predictive performance and low privacy risk.

As shown in Figure 9, our proposed SDP method consistently achieves a lower SSIM at the same level of prediction accuracy than MSDP and PriMIA. For instance, at an accuracy level of 0.7, SDP achieves an SSIM value of approximately 0.22, while MSDP and PriMIA have significantly higher leakage. This indicates that our method maintains stronger privacy protection without compromising utility. In contrast, PriMIA exhibits steep increases in the SSIM once accuracy surpasses 0.6, suggesting a poor balance between privacy and learning performance. A similar pattern can be observed in Figure 10. Across the entire accuracy range, the SSIM of SDP is consistently below the baseline methods. Even when achieving high prediction accuracy near 0.9, our method maintains lower SSIM values than MSDP and PriMIA. This demonstrates that the privacy-preserving effect of our sensitivity-aware mechanism generalizes well to high-dimensional and colored medical images. Taken together, these trade-off curves confirm that the proposed SDP framework achieves a more favorable balance between privacy protection and model performance. By calibrating noise according to data sensitivity, our method prevents excessive leakage while retaining high predictive accuracy. These properties make SDP a robust and adaptable solution for federated learning scenarios, particularly in privacy-sensitive domains such as medical imaging.

**Figure 11:**
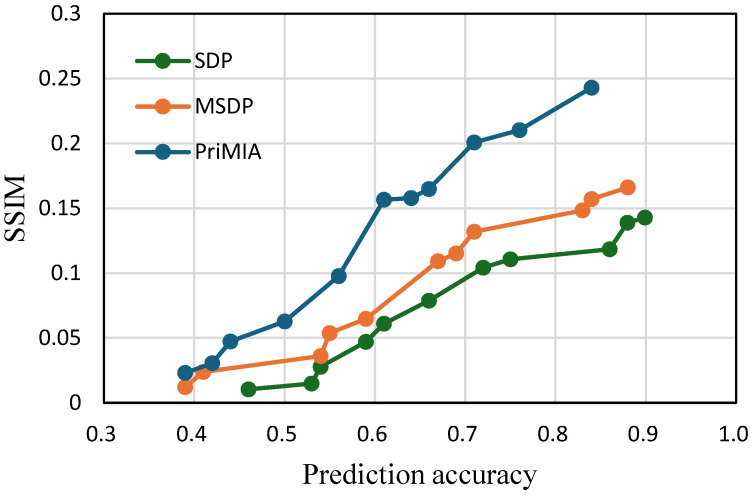
The trade-off between accuracy and privacy with a batch size of 32 (COVID-19 dataset).

**Figure 12:**
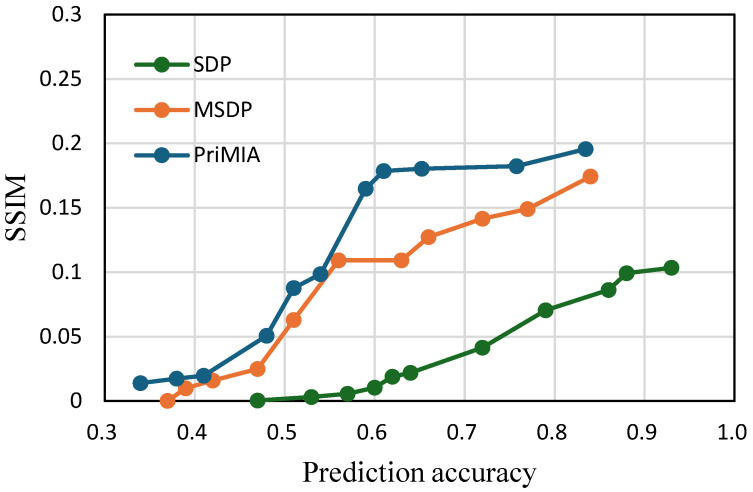
The trade-off between accuracy and privacy with a batch size of 32 (REA dataset).

**Impact of batch size on privacy–utility trade-off.** All previous experiments were conducted with a batch size of one, which corresponded to the worst-case scenario for gradient inversion attacks (GIAs). This setting maximizes the attacker’s ability to reconstruct individual data samples and therefore provides a rigorous benchmark for evaluating privacy-preserving mechanisms. To investigate how the proposed method behaves under more realistic federated learning conditions, we further evaluated all methods with a batch size of 32. The results are presented in Figure 11 for the COVID-19 dataset and Figure 12 for the REA dataset.

As shown in both figures, increasing the batch size significantly reduces privacy leakage with all methods, evidenced by the lower SSIM values compared to the batch size one setting. This observation aligns with previous findings in the literature [12], which suggest that GIA becomes less effective when gradients are computed over multiple data points. Even under these milder attack conditions, our proposed SDP framework maintains a clear advantage in balancing privacy and utility. In Figure 11, we can observe that SDP consistently yields lower SSIM values than both MSDP and PriMIA at comparable levels of prediction accuracy. For instance, when the accuracy reaches 0.8, the SSIM of SDP remains below 0.15, whereas PriMIA exceeds 0.25. A similar pattern is evident in Figure 12, where SDP maintains substantially lower SSIM values across the entire accuracy range, particularly when model utility is high. These results demonstrate that SDP continues to provide strong privacy protection while enabling competitive or superior accuracy even when the threat of GIA is diminished by larger batch sizes.

The effectiveness of SDP in this setting stems from its sensitivity-aware noise allocation strategy. Unlike traditional DP methods that apply uniform noise regardless of data vulnerability, SDP dynamically adjusts the noise level for each sample based on its estimated sensitivity. When the batch size increases and the overall attack surface is reduced, SDP naturally reduces the amount of noise added to most samples. This leads to improved model utility while still offering targeted protection for the few samples that remain vulnerable. This adaptive behavior enables SDP to maintain a well-balanced trade-off between privacy and performance under varying training configurations, highlighting its practicality and robustness in real-world federated learning deployments.

## 7. Discussion

### 7.1. Generalizing SDP Across Data Modalities

Although this study focused on medical imaging datasets such as chest X-rays and retinal fundus images, the proposed sensitivity-aware differential privacy (SDP) framework is not inherently limited to image data. In our experiments, we adopted the structural similarity index measure (SSIM) to quantify privacy leakage by measuring the degree of similarity between reconstructed and original images. However, the underlying design of SDP is inherently adaptable and can be extended to other data modalities by incorporating alternative similarity measures for sensitivity estimation. For textual data, semantic similarity can be computed using cosine similarity applied to word or sentence embeddings such as Word2Vec or BERT. This allows the framework to assess how much sensitive information might be exposed through reconstruction. In tabular data, distance-based metrics such as the Kullback–Leibler (KL) divergence or Mahalanobis distance may be more suitable, as they capture shifts in feature distributions that may reflect privacy leakage. By allowing for the integration of modality-specific similarity metrics, the SDP framework can generalize effectively to non-visual data types. This flexibility makes it well suited for a broad spectrum of privacy-sensitive federated learning applications, including natural language processing and electronic health record analysis, where maintaining both rigorous privacy guarantees and high task-specific utility is essential.

### 7.2. Extending SDP with Cryptographic Techniques

While the proposed sensitivity-aware differential privacy (SDP) mechanism effectively reduces privacy leakage by adaptively calibrating noise based on sample-level sensitivity, it can be further enhanced through integration with cryptographic techniques such as secure aggregation. Secure aggregation protocols are designed to prevent the central server from accessing individual model updates by revealing only their aggregated form. However, these protocols do not eliminate the risk of information leakage from the global model itself, particularly in scenarios where an adversary may infer sensitive content from the aggregated parameters or where multiple clients engage in collusion. The SDP framework addresses this limitation by ensuring that each client’s update is already differentially private before aggregation. This approach provides privacy protection at the local data level, independent of the aggregation process. When combined with secure aggregation, SDP introduces an additional layer of defense that remains effective even if the server is compromised or if clients act maliciously in coordination. This dual-layer privacy architecture is especially advantageous in multi-institutional federated learning environments, where the participating entities often have varying privacy requirements and limited trust in one another. In such settings, the integration of SDP with secure aggregation enables end-to-end privacy protection: local differential privacy is applied on the client side, while secure aggregation safeguards communication at the system level. This combination enhances the overall robustness and deployability of federated learning frameworks in sensitive domains such as healthcare and finance.

## 8. Conclusions

In this paper, we introduce a novel privacy notion, sensitivity-aware differential privacy (SDP), which provides varying degrees of privacy protection for different data while enhancing model performance. Unlike previous methods, SDP determines the sensitivity levels of training data based on privacy risks under real-world attacks. To implement this notion in federated medical imaging, we propose a privacy mechanism that evaluates the sensitivity of each data sample using gradient inversion attacks and customizes the noise accordingly. Moreover, this method seamlessly extends to complex multi-attack scenarios, effectively quantifying comprehensive privacy risks and dynamically adjusting protection measures. Extensive experiments demonstrate that this sensitivity-driven approach achieves an optimal balance between privacy protection and utility. Furthermore, SDP is not limited to medical imaging and can be generalized to other domains where data exhibit varying privacy risks. 

## Figures and Tables

**Table 1:** Comparison of DP methods.

Study	Methodology	Addressed Gap	Limitations
[21]	DPSGD	Prevents information leakage in FL	Uniform noise may degrade utility
[27]	Personalized DP	Considers the sensitivity of different users	Ignores varying data sensitivities
[28]	Selective-DP	Protects only the sensitive samples	Lacks consideration of real-world attacks
Our method	Sensitivity-aware DP	Dynamically adjust noise based on measured sensitivity to privacy attacks	Future work considers different modal data and the combination with cryptography

**Table 2:** Performance comparison under different privacy budgets.

Methods	ϵ=1		ϵ=5		ϵ=10	
	**Accuracy**↑	**SSIM**↓	**Accuracy**↑	**SSIM**↓	**Accuracy**↑	**SSIM**↓
[21]	0.42	0.08	0.56	0.35	0.86	0.54
[29]	0.45	0.09	0.60	0.32	0.87	0.53
Our method	0.59	0.06	0.71	0.26	0.90	0.47

## Data Availability

The datasets used in this study are publicly available. The COVID-19 dataset can be accessed at https://www.kaggle.com/datasets/anasmohammedtahir/covidqu (accessed on 1 September 2023), and the REA dataset is available at https://zenodo.org/records/5793241 (accessed on 1 September 2023). Additionally, the code used for implementation, model training, and evaluation is available upon request. Researchers interested in reproducing our results or extending this work are encouraged to contact the authors for access to the source code and further details.

## Abbreviations

The following abbreviations are used in this manuscript:

FL	Federated learning
DP	Differential privacy
GIA	Gradient inversion attacks
AI	Artificial intelligence
SDP	Sensitivity-aware differential privacy
DPSGD	Differentially private stochastic gradient descent
SSIM	Structural similarity index measure
REA	Rotterdam EyePACS AIROGS
DLG	Deep leakage from gradient
LDP	Local differential privacy
